# Supplementary material for: Evaporation-induced sintering of liquid metal droplets with biological nanofibrils for flexible conductivity and responsive actuation
Source: Nat Commun. 2019 Aug 5;10:3514. doi: 10.1038/s41467-019-11466-5 (PMC6683165; doi:10.1038/s41467-019-11466-5)
Supplement: Supplementary file 1 — Supplementary Information [file 41467_2019_11466_MOESM1_ESM.pdf]

# Supplementary Information

## Evaporation-induced sintering of liquid metal droplets with biological nanofibrils for flexible conductivity and responsive actuation

**Li et al**

## Supplementary Figures

### Supplementary Figure 1

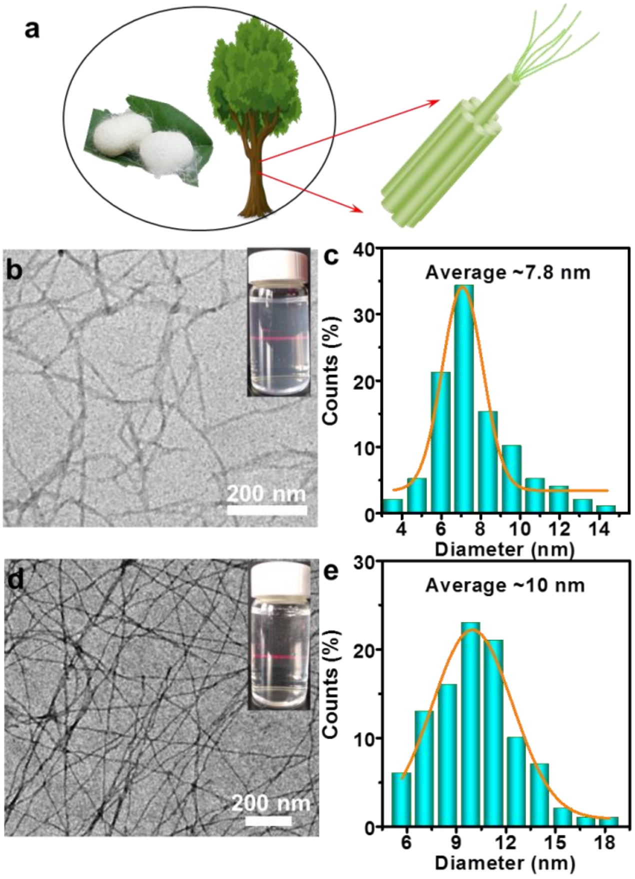


**Supplementary Figure 1. Synthesis of biological NFs.** (**a**) Schematic illustration of synthesizing biological NFs from biomass via liquid exfoliation. TEM images and diameter histogram of amyloid NFs (**b & c**) and silk NFs (**d & e**). The inset gives visual observation of corresponding aqueous suspensions (0.1 wt%).

**Supplementary Figure 2**

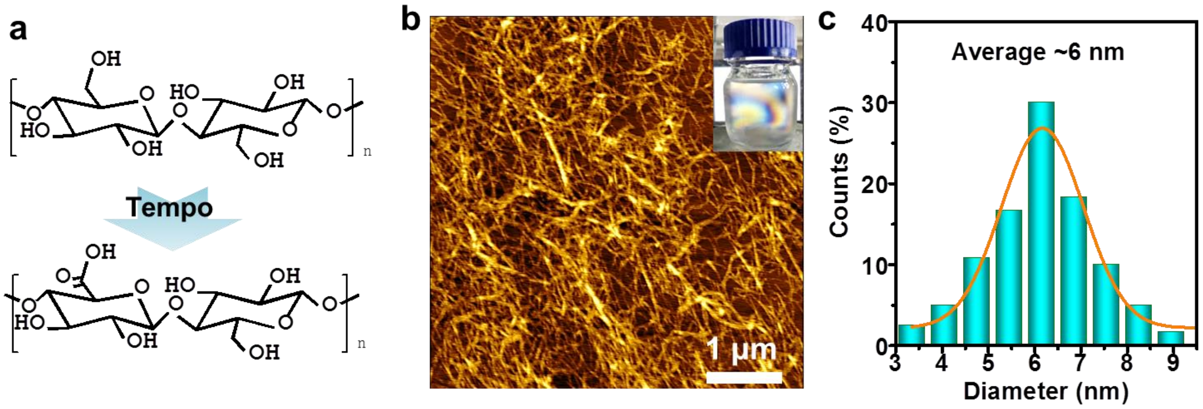


**Supplementary Figure 2. Chemical and physical structure of CNFs.** (**a**) Chemical structure of cellulose before and after TEMPO-mediated oxidation. Atomic force microscope image (**b**) and diameter histogram (**c**) of CNFs. Atomic Force Microscope (AFM) image was performed in the tapping mode on an Aglient 5400 equipped with silicon nitride cantilevers (Bruker) at a scan rate of 1Hz.

**Supplementary Figure 3**

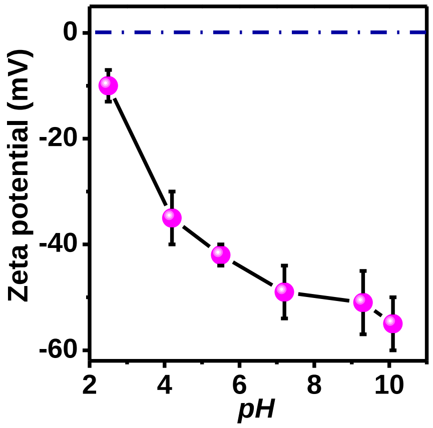


**Supplementary Figure 3.** Zeta potential variation of CNFs in aqueous suspension ($\phi_{CNF}$ = 0.2 wt%) at different pHs.

**Supplementary Figure** 4

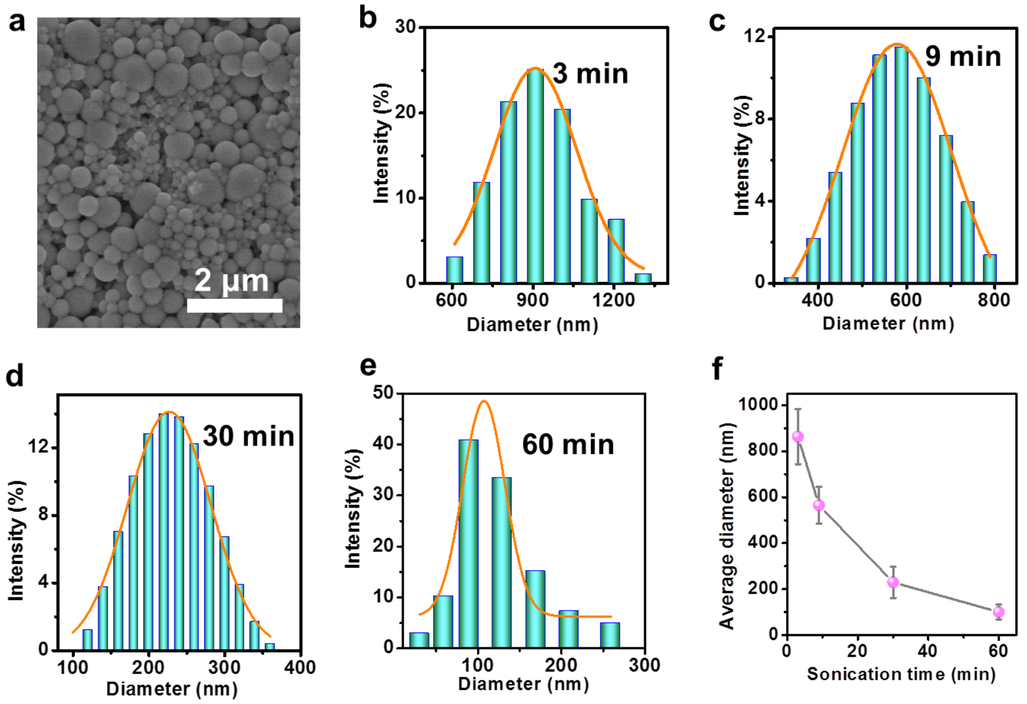


**Supplementary Figure 4. Variation of EGaIn droplet size versus ultrasonic time.** (**a**) Typical SEM image of EGaIn droplets after centrifuging at 6000 rpm for 15 min. 150 mg EGaIn in 15 mL water, $\phi_{CNF}$=0.2 wt%, ultrasonic ~60 min, power 300 W (without further specification). Histogram variation of EGaIn droplet diameter (**b-e**) and corresponding average size (**f**) at different ultrasonic time.

**Supplementary Figure 5**

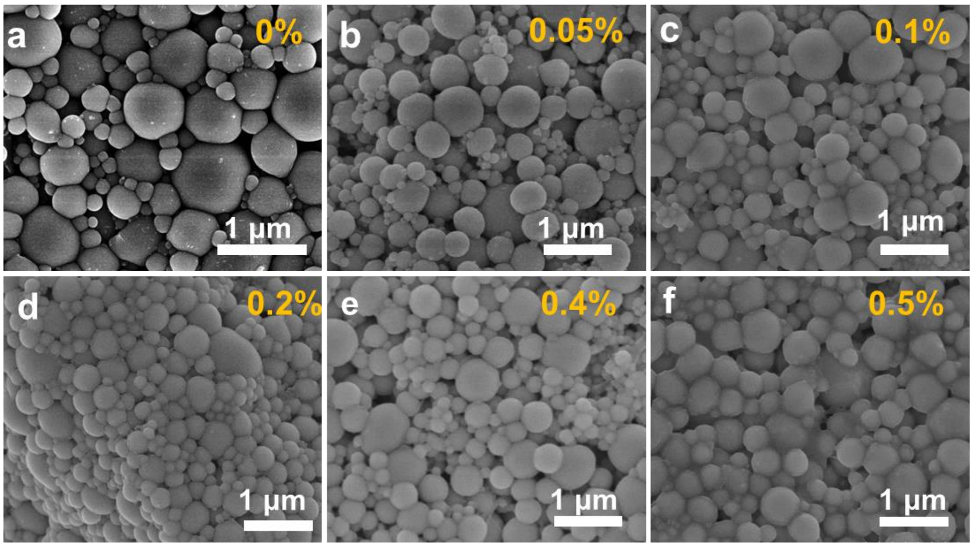


**Supplementary Figure 5. Variation of EGaIn droplet size versus CNFs after centrifugation.** SEM images of EGaIn droplets produced with indicated concentrations of CNFs: (**a**) 0 wt%, (**b**) 0.05 wt%, (**c**) 0.1 wt%, (**d**) 0.2 wt%, (**e**) 0.4 wt% and (**f**) 0.5 wt%. Centrifugation at 6000 rpm for 15 min.

**Supplementary Figure 6**

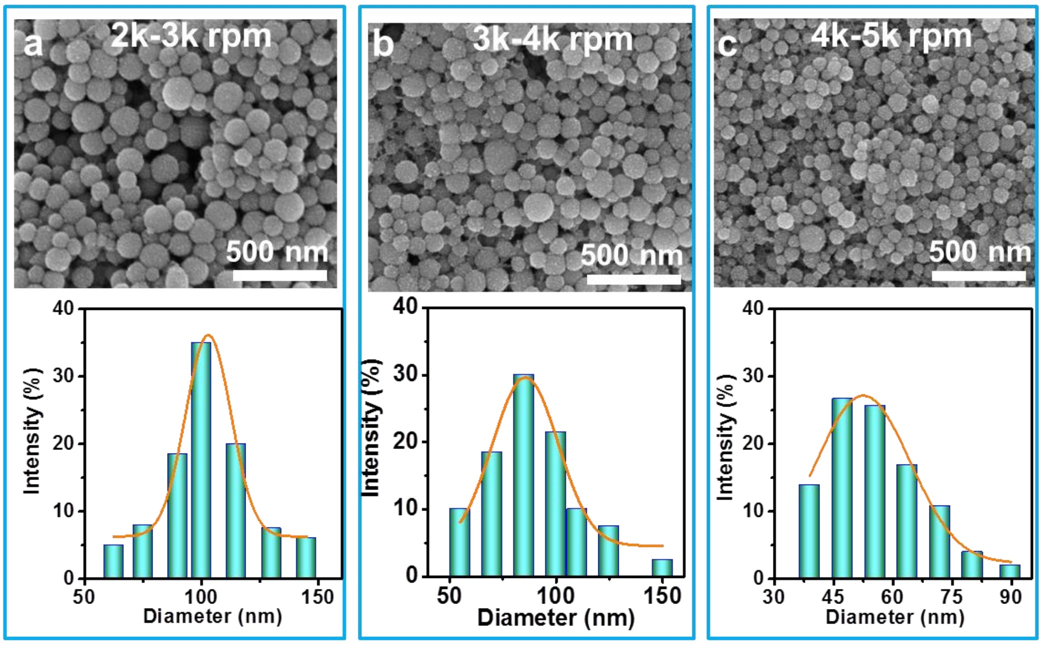


**Supplementary Figure 6. Sizing EGaIn droplets with centrifugation.** SEM images and size histogram of EGaIn droplets after centrifugation with indicated spinning rates: (**a**) 2k–3k, (**b**) 3k–4k, and (**c**) 4k–5k rpm.

**Supplementary Figure 7**

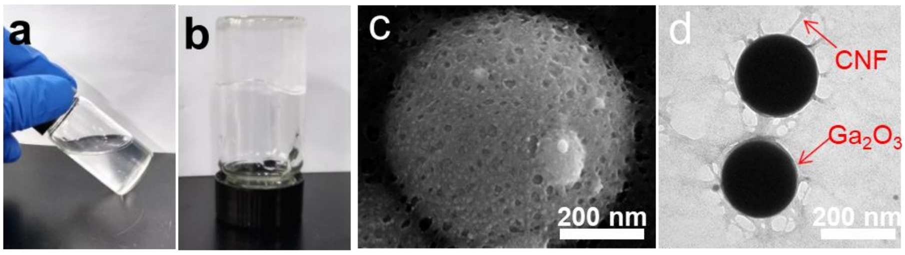


**Supplementary Figure 7. Gelation of CNFs with $Ga^{3+}$.** CNFs dispersion ($\phi_{CNF}$ = 0.2 wt%) before (**a**) and after (**b**) gelation with 0.78 mM $Ga^{3+}$. Typical SEM (**c**) and TEM image (**d**) of EGaIn droplets encapsulated in oxide shell and wrapped with a certain amount of CNFs on their surface.

**Supplementary Figure 8**

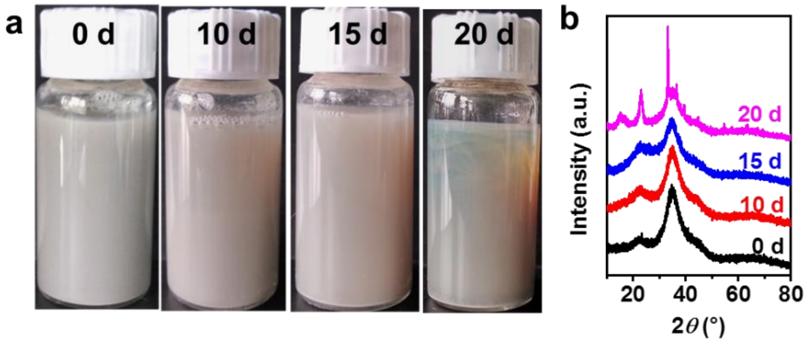


**Supplementary Figure 8. Stability of EGaIn droplets after storing for different time periods.** Visual observation (**a**) and evolution of XRD patterns (**b**) of EGaIn droplets produced in CNFs suspension after storing for indicated periods of time with $N_2$ purge.

**Supplementary Figure 9**

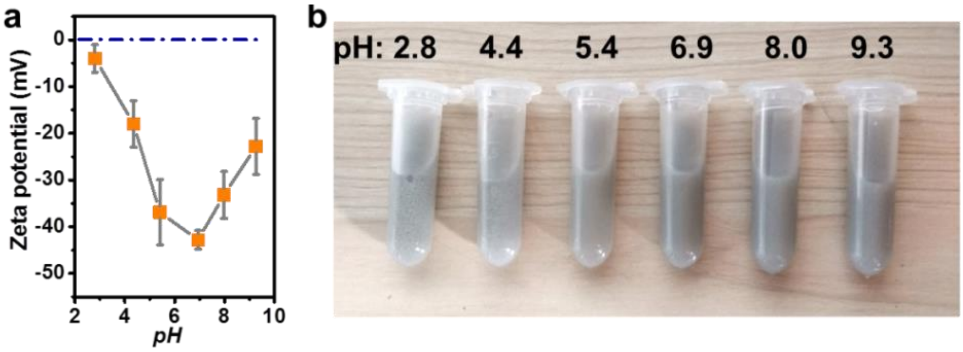


**c**

$$pH < 7:\ 2Ga + 6H^+ = 2Ga^{3+} + 3H_2\uparrow$$

$$pH = 7:\ 2Ga + 4H_2O = 2GaOOH + 3H_2\uparrow$$

$$pH > 7:\ 2Ga + 6OH^- + 6H_2O = 2[Ga(OH)_4]^- + 3H_2\uparrow$$

$$2Ga + 2H_2O + O_2 = 2GaOOH + H_2\uparrow$$

$$4Ga + 2H_2O + 3O_2 = 4GaOOH$$

**Supplementary Figure 9. Stability of EGaIn droplets at different pHs.** Zeta potential variation (**a**) and visual observation (**b**) of EGaIn droplets produced in CNFs suspension at different pHs. (**c**) Reactions of EGaIn droplets in water at different pHs.

**Supplementary Figure 10**

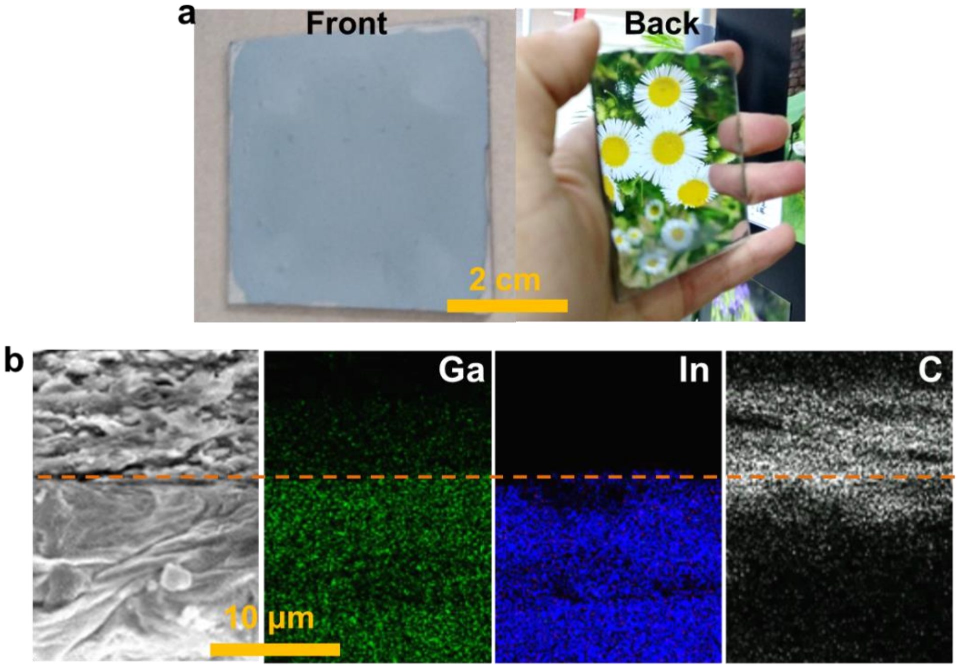


**Supplementary Figure 10. Janus properties of the resultant free-standing film.** (**a**) Visual observation of top and bottom sides of EGaIn coating on glass. Free-standing Janus film could be peeled off from this coating. (**b**) Cross-sectional SEM image and Ga, In, C elemental mapping of Janus film.

**Supplementary Figure 11**

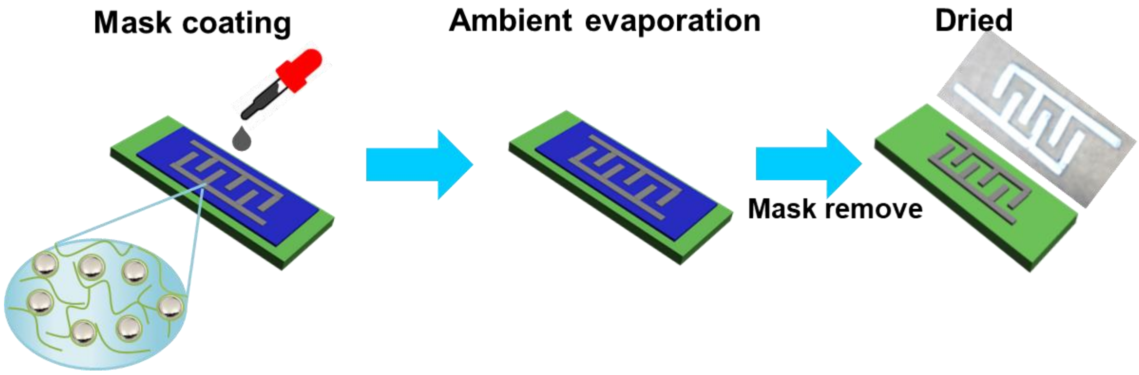


**Supplementary Figure 11.** Procedures of mask-assistant drop-coating and evaporation-induced sintering of EGaIn droplets.

**Supplementary Figure 12**

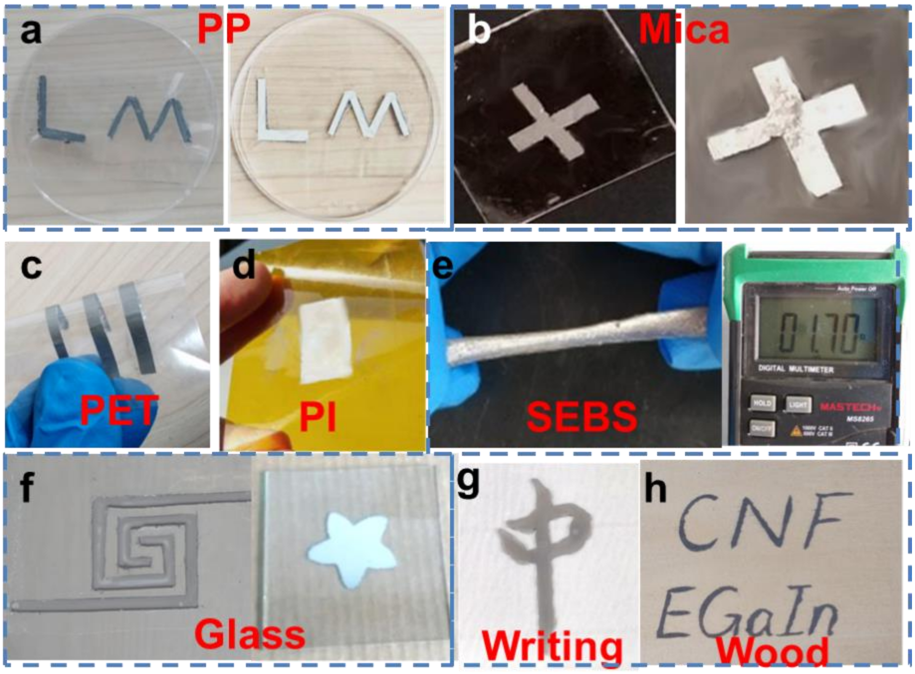


**Supplementary Figure 12. Visual observation of pattern or coating layers on indicated substrates.** Top (left) and bottom sides (right) on PP (**a**) and mica (**b**) by drop-casting. Mask depositing on PET (**c**), Polyimide (PI) (**d**) and glass (**f**). (**e**) Drop-casting on SEBS. Hand-writing on glass (**g**) and wood (**h**).

**Supplementary Figure 13**

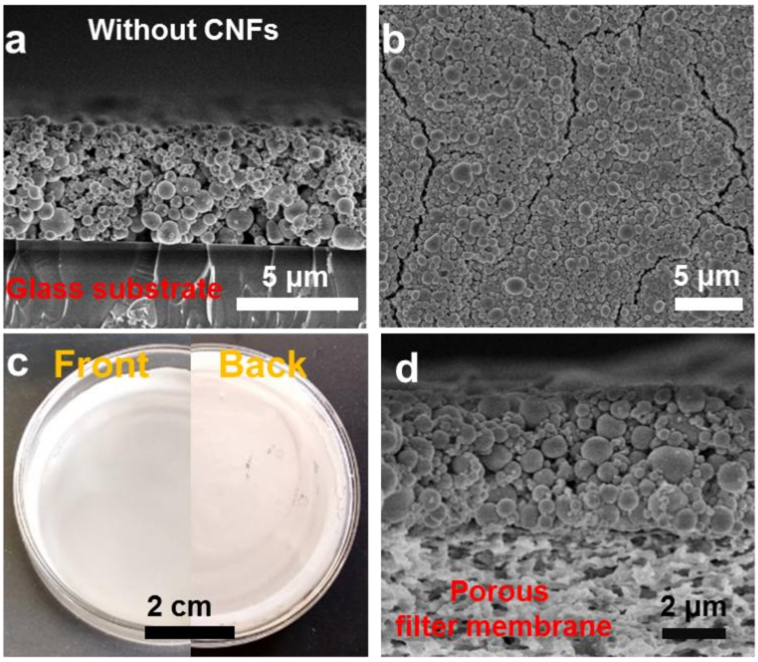


**Supplementary Figure 13. Deposits of EGaIn droplets in absence of NFs.** SEM image (**a**) and cracks (**b**) when depositing EGaIn droplets with absence of NFs. (**c**) Optical images of top and bottom surfaces when depositing EGaIn droplets with absence of NFs. (**d**) SEM image when filtering suspension of EGaIn droplets without NFs. Membrane pore size: 220 nm.

**Supplementary Figure 14**

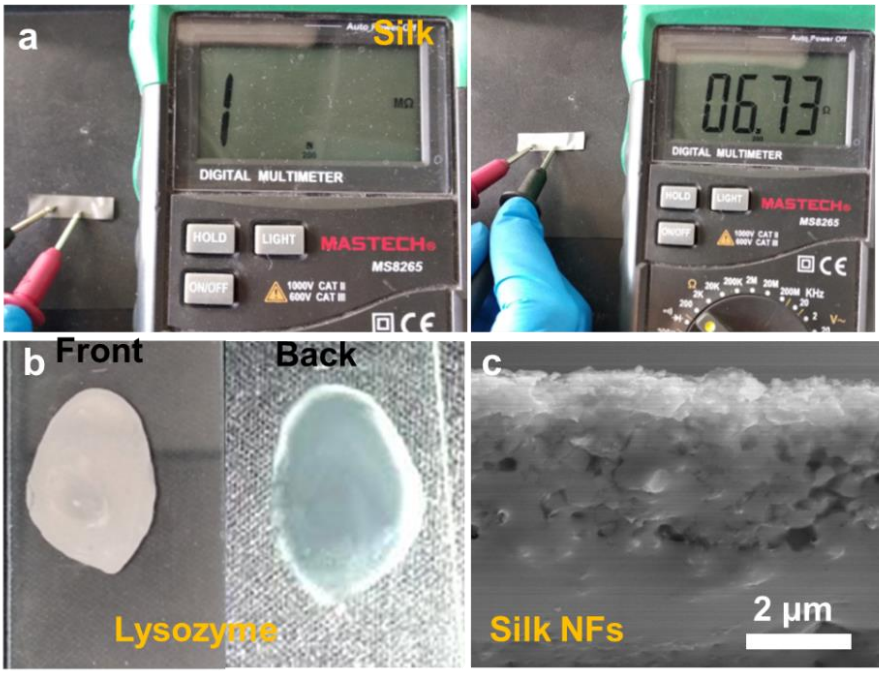


**Supplementary Figure 14. Sintering of EGaIn droplets with other biological nanofibrils except CNFs.** Optical images of EGaIn coating in presence of silk NFs (**a**) and amyloid NFs (**b**). (**c**) Cross-sectional SEM image of EGaIn coating in presence of silk NFs.

**Supplementary Figure 15**

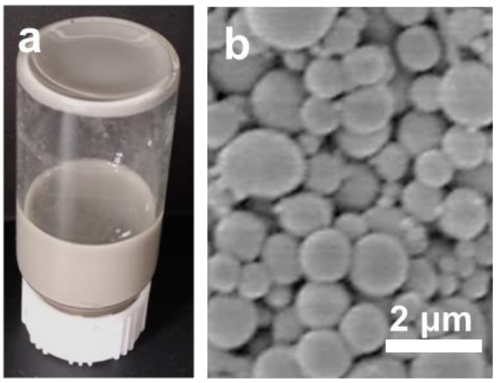


**Supplementary Figure 15**. **Sedimentation phenomenon of EGaIn droplets dispersion during evaporation.** (**a**) Optical image of evaporation-induced sedimentation of EGaIn droplets with presence of CNFs. (**b**) SEM image of EGaIn droplets after sedimentation for 6 h, indicating chemical stability during drying.

**Supplementary Figure 16**

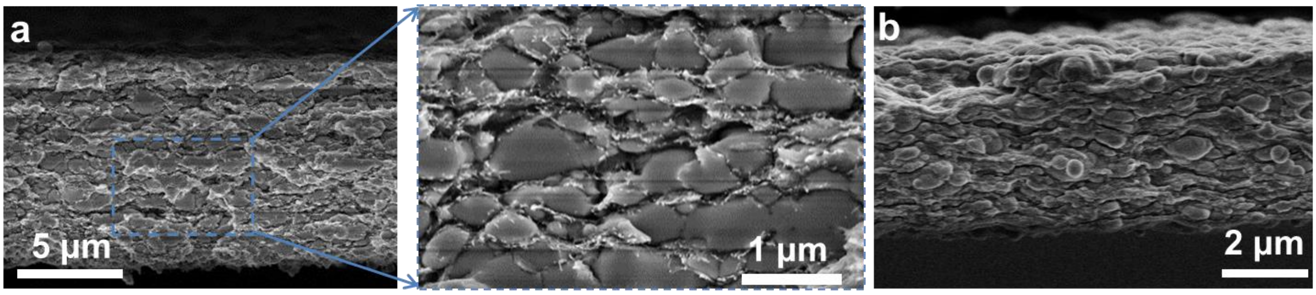


**Supplementary Figure 16. SEM image of EGaIn droplets deposit with rapid solvent removal without sedimentation.** (**a**) Cross-sectional SEM image after filtering suspension of EGaIn droplets with CNFs. Membrane pore size: 220 nm. (**b**) Cross-sectional SEM image after rapidly drying EGaIn droplets with CNFs.

**Supplementary Figure 17**

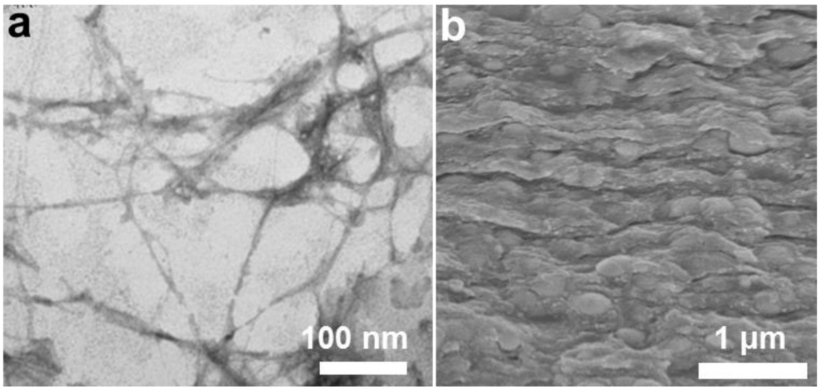


**Supplementary Figure 17. Evidence for the two distinct layers.** (**a**) TEM image of centrifugate of EGaIn-rich layer dissolving in 0.1 M HCl. (**b**) SEM image of CNFs-rich layer.

**Supplementary Figure 18**

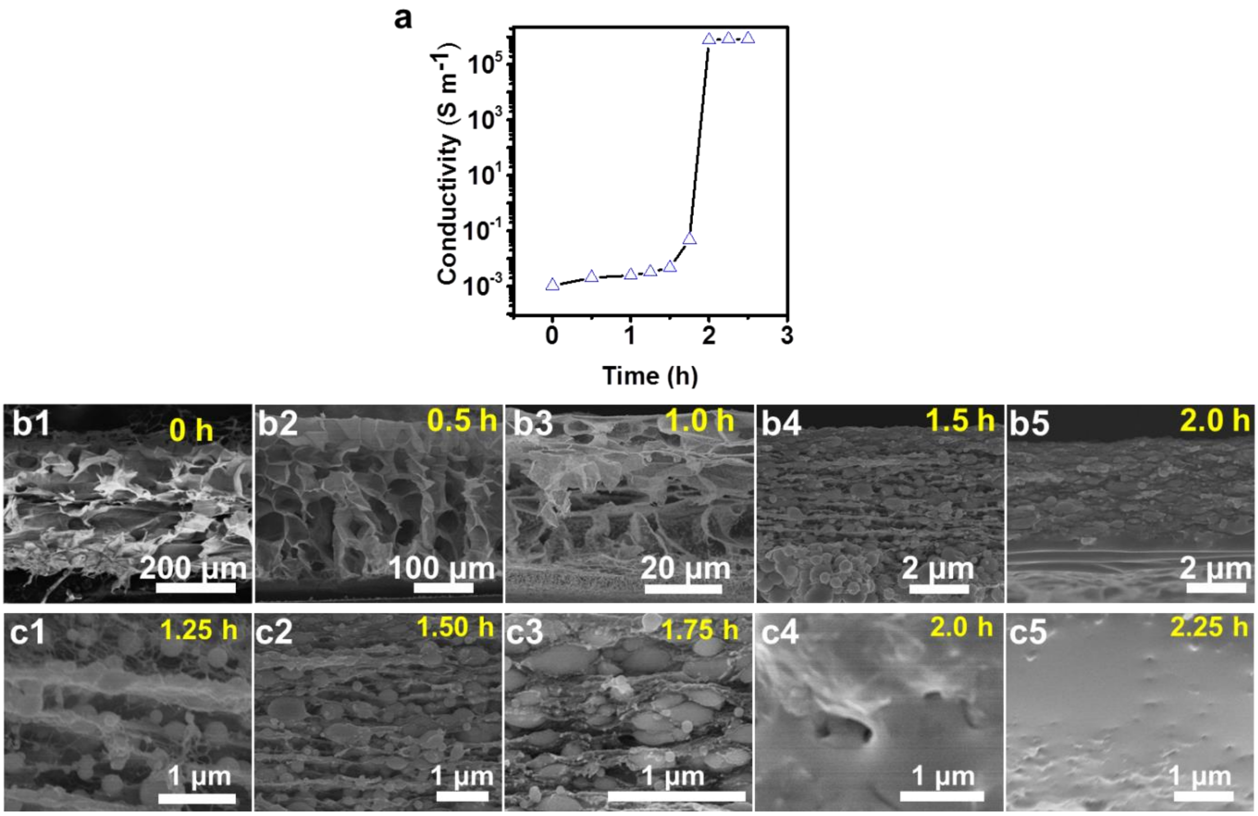


**Supplementary Figure 18. Conductivity and freeze-dried SEM image variation versus time during drying of the EGaIn droplets suspension.** (**a**) Conductivity variation versus time during drying suspension of EGaIn droplets on glass. Suspension volume of EGaIn droplets: 0.5 mL; $\phi_{CNF}$: ≤0.2 wt%; Glass substrate: 1.5 × 1 cm$^2$. Before solvent evaporation, only a low ionic conductivity was detected in the fluid layer of EGaIn droplets. During solvent evaporation, the capillary force may be high enough to sinter EGaIn droplets and produce a conductivity increase. (**b**) Cross-sectional SEM image when drying suspension of EGaIn droplets on glass for indicated periods of time. Each specimen underwent a freeze-drying process before SEM characterization. (**c**) SEM images for characterization of sintering process of EGaIn droplets during solvent evaporation. Before solvent evaporation, EGaIn droplets was apt to precipitate first on the bottom due to its large density

of ~6 g cm$^{-3}$, while free CNFs which were not attached on EGaIn droplets tended to keep colloidallly stable in the suspension (Supplementary Figure 3, 8 and 9). At the final stage of solvent evaporation, capillary force was high enough to sinter EGaIn droplets and formed a conductive EGaIn-rich layer. Part of CNFs would stay over the EGaIn-rich layer and dry into a CNFs-rich layer. To be noted, part of EGaIn droplets may had the smaller size and attach a large amount of CNFs, which would show higher colloidal stability and thus stay in the CNFs-rich layer after completely drying.

**Supplementary Figure 19**

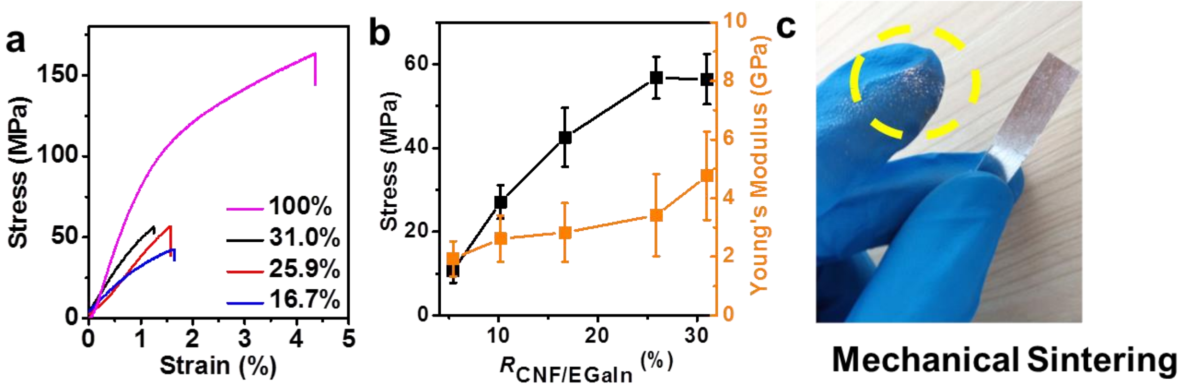


**Supplementary Figure 19. Optimizing the mechanical properties of free-standing Janus film by varying CNFs concentration.** (**a & b**) Mechanical properties of free-standing Janus film with different CNFs compositions. (**c**) Endurance of EGaIn layer to mild rub which was produce without biological NFs.

**Supplementary Figure 20**

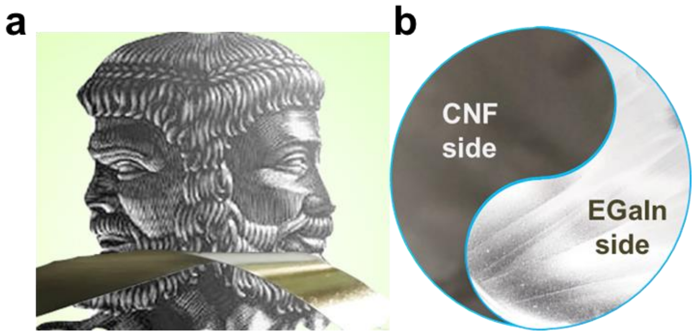


**Supplementary Figure 20. Optical observation of the free-standing films.** Their distinct CNFs-rich and EGaIn-rich sides are just like the two faces of Janus in Roman mythology (**a**) and two poles of Tai Chi in Chinese philosophy (**b**).

**Supplementary Figure 21**

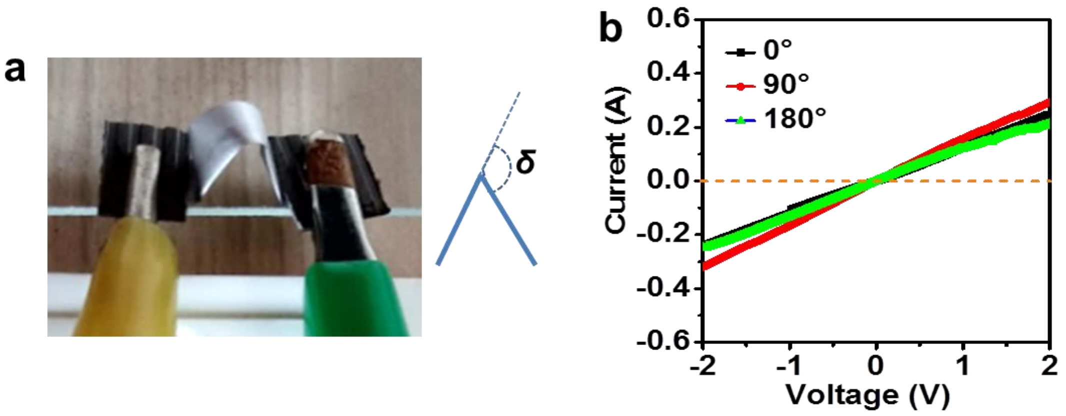


**Supplementary Figure 21. Flexible conductivity of Optical image EgaIn-rich coating.** Optical image (**a**) and current-voltage curve (**b**) when bending conductive EGaIn layer (thickness of 10 μm) on SEBS. EGaIn layer was produced by evaporation-induced sintering. Current-voltage curves were recorded on a CHI660E electrochemical workstation (CH Instrument, Shanghai, China) with a scan rate of 0.1 V $s^{-1}$.

**Supplementary Figure 22**

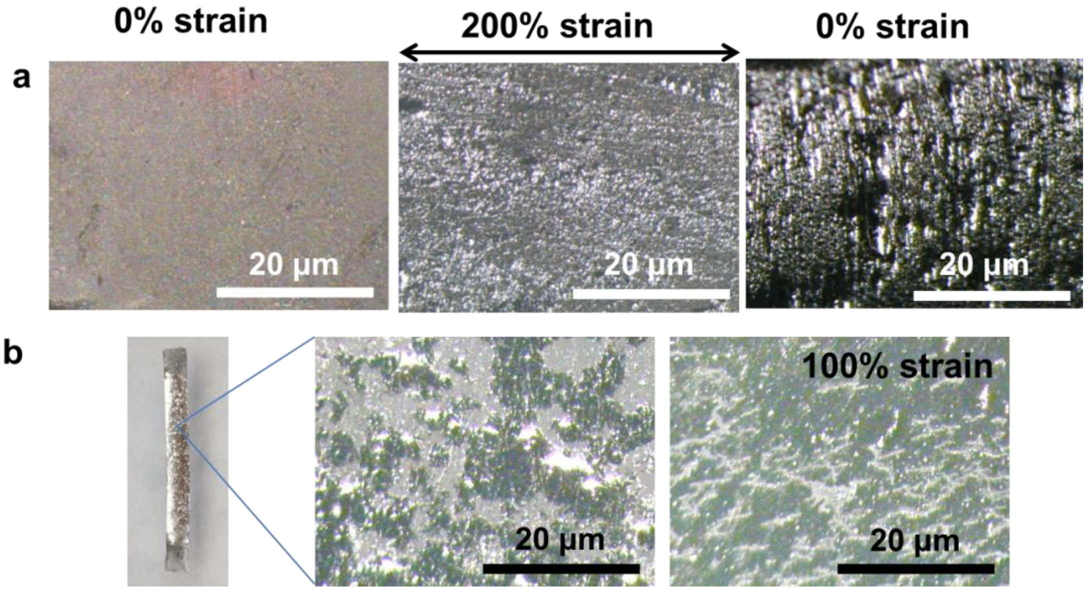


**Supplementary Figure 22. Affinity of evaporation-sintered EGaIn layer with SEBS under stretch.** Optical images of EGaIn layer (thickness of 10 μm) on SEBS at different strains. EGaIn layer were produced by evaporation-induced sintering (**a**) and depositing bulk LM (thickness of ~50 μm) (**b**).

**Supplementary Figure 23**

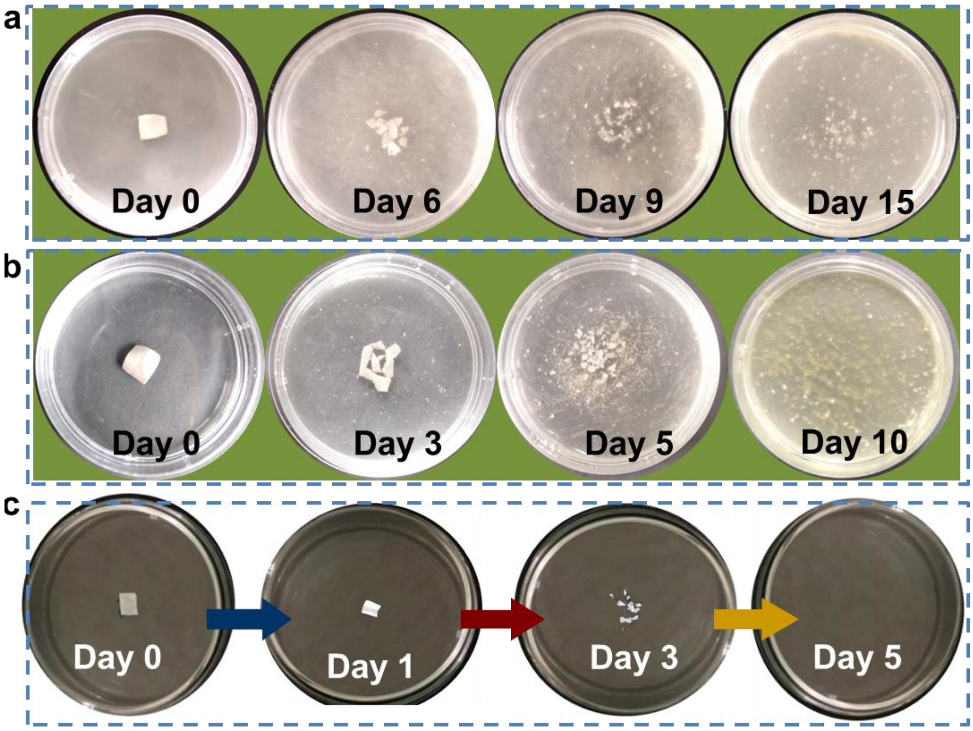


**Supplementary Figure 23. Degradability of the EGaIn Janus film.** (**a** & **b**) Biological degradation of EGaIn Janus film for indicated periods of time in soil extract with (**a**) and without (**b**) pre-cultivation in 5 mg $mL^{-1}$ glucose for 24 h at 30 °C. (**c**) Chemical degradation of EGaIn Janus film for indicated periods in 1 M NaOH.

**Supplementary Figure 24**

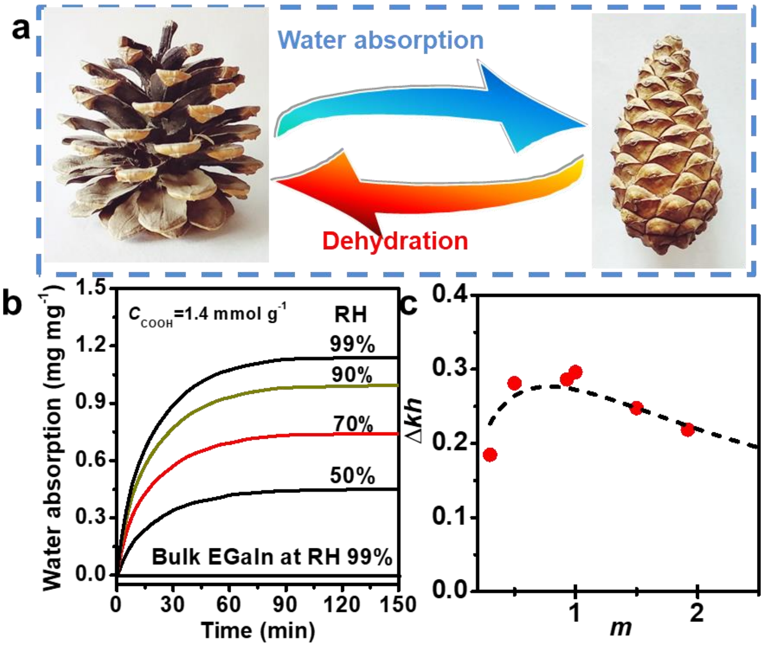


**Supplementary Figure 24. Optimizing biomimetic actuating behavior of Janus film.** (**a**) Cyclic switch of dehydration and water absorption for reversible opening movement of pine cones.[1] (**b**) Water uptake of CNFs and bulk EGaIn at different RH values. (**c**) Optimizing actuating behavior of Janus film (40 × 10 $mm^2$) with bilayer thickness. According to the theory for bimetallic thermostats:[2]

$$\Delta\kappa h \sim \frac{6(1+m)^2}{6(1+m)^2+(1+mn)(m^2+1/mn)} \qquad (1)$$

where Δκ is the curvature change of the bilayer, $h_{EGaIn}$ and $h_{CNF}$ are the thickness of the constituent EGaIn and CNFs layers (total thickness $h = h_{EGaIn} + h_{CNF}$), $E_{EGaIn}$ and $E_{CNF}$ are their Young's moduli, and $m = h_{EGaIn}/h_{CNF}$, $n = E_{EGaIn}/E_{CNF}$. A maximum actuation curvature could be obtained when the thickness of EGaIn layer is ~0.9 times that of the CNFs layer.

**Supplementary Figure 25**

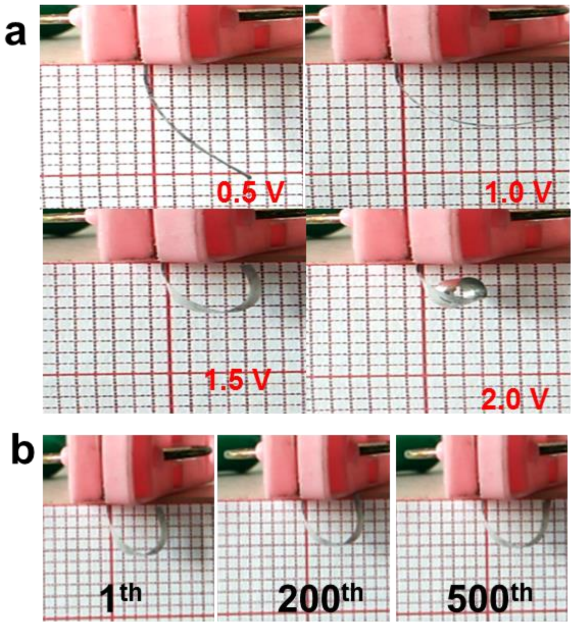


**Supplementary Figure 25. Voltage-induced actuating of the Janus film.** Visual observation of actuating behavior of Janus film at indicated voltages (**a**) and for indicated actuating cycles at voltage of 1.5 V (**b**).

**Supplementary Figure 26**

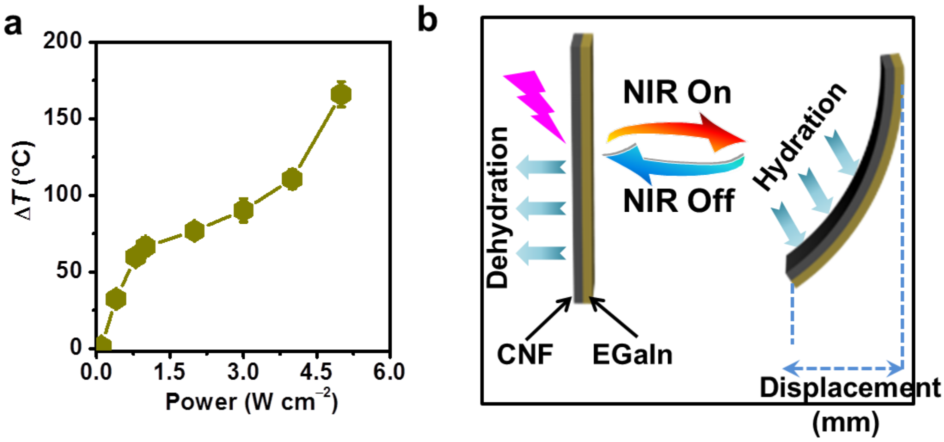


**Supplementary Figure 26. Photo-induced actuating mechanism of the Janus film.** (**a**) Photo-thermal effect of Janus film exposing to different NIR power densities. (**b**) Schematic illustration of actuating mechanism. Dehydrating CNFs-rich layer with its photo-thermal effect. Displacement was defined as indicated.

**Supplementary Figure 27**

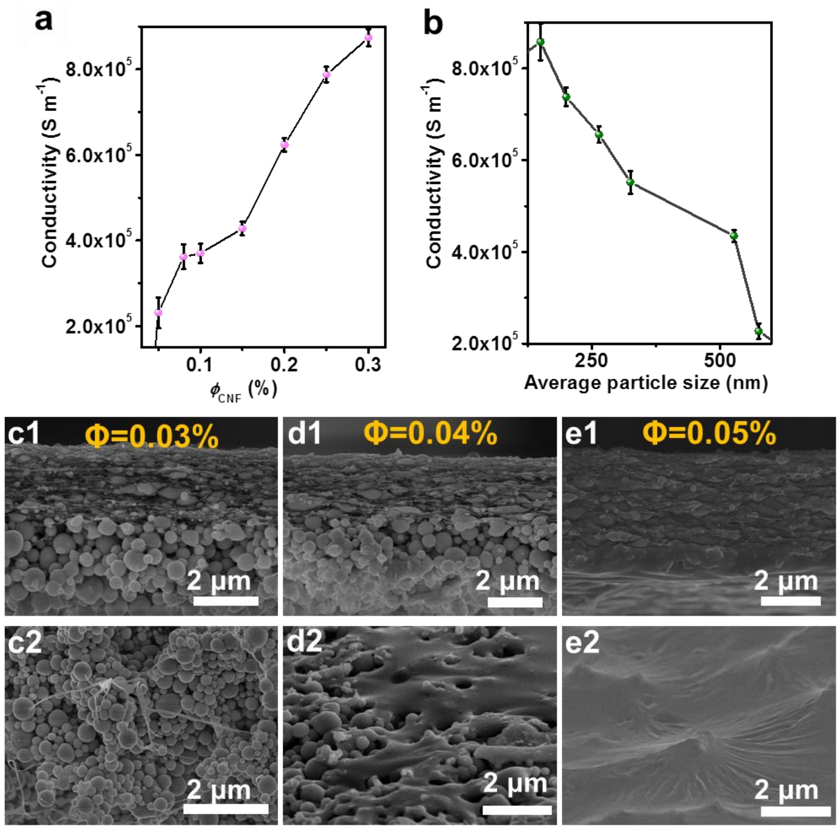


**Supplementary Figure 27. Conductivity and freeze-dried SEM image variation versus CNFs concentration during drying of the EGaIn droplets suspension.** (**a** & **b**) Effects of CNFs concentration (**a**) and droplet size (**b**) on evaporation-induced sintering evaluated by conductivity of EGaIn-rich side. EGaIn droplet size: ~120 nm. (**c-e**) Cross-section (Top) and back-surface (Bottom) SEM images of EGaIn layers producing with different CNFs concentrations. $\phi_{CNF}$: 0.03 wt% (**c**), 0.04 wt% (**d**), 0.05 wt% (**e**) With a low CNFs concentration (e.g., $\phi_{CNF} \leq 0.03$ wt%), no trace of sintering was detected, and thus resulted in an ultralow conductivity due to the presence of insulating oxide shells of EGaIn droplets. At $\phi_{CNF} > 0.04$ wt%, EGaIn droplets started to be sintered and produce a conductivity increase. At $\phi_{CNF} = 0.05$ wt%, EGaIn droplets were sintered and form a relatively homogenous conductive layer with a metallic conductivity up to $10^5$ S $m^{-1}$.

**Supplementary Figure 28**

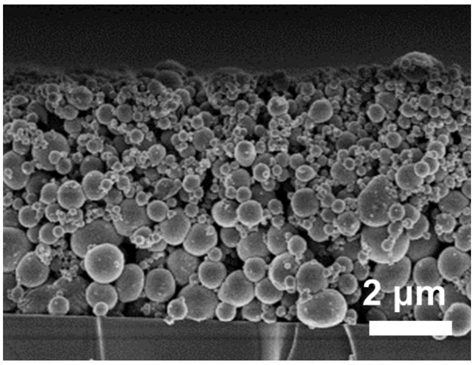


**Supplementary Figure 28. Cross-sectional SEM image after depositing suspension of EGaIn droplets on glass substrate.** EGaIn droplets were produced with CNFs ($\phi_{CNF}$ = 0.2 wt%) followed by three times' centrifugation at 6000 rpm for 15 min to remove free CNFs.

## Supplementary Notes

### Supplementary Note 1

The capillary force arising from the Laplace pressure can be estimated analytically by the following equation:[3]

$$F = 2\pi\gamma\alpha \cos\theta / [1 + H/2d] \qquad (2)$$

where $H$ is the shortest distance between the spheres; $d$ is the immersion length of the sphere and calculated by $d = \left({}^{H}/_{2}\right) \times [-1 + \sqrt{1 + 2V/(\pi\alpha H^2)}]$, where $V$ is the volume of liquid bridge.

The pressure difference cross the liquid surfaces can be calculated as:

$$F/\pi[\alpha^2 - (\alpha - d)^2] \qquad (3)$$

If assuming $\alpha$ = 60 nm, $V = 10^3$ nm$^3$, $\gamma$ = 72 mN m$^{-1}$, $H$ = 5 nm and $\theta$ = 5º (For simplification, the contact angles of TEMPO-CNFs was used.[4]). The compressive pressure is 13.7 MPa. Without the presence of CNFs, if the contact angle of bulk EGaIn with $Ga_2O_3$ surfaces (~ 86º) was used, the capillary force was calculated as 0.9 MPa, which is much lower than the critical sintering pressure ~5 MPa.[5]

**Supplementary Methods**

**Preparation of biological NFs.** TEMPO-mediated oxidation was applied to synthesize carboxylated CNFs according to a well-established procedure.[6] Typically, hardwood cellulose pellicles (3 g) were suspended into water (300 mL) with TEMPO (0.048 g) and NaBr (0.3 g) under vigorous stirring. NaClO solution (9.3 g) was slowly added under gentle stirring. The pH of the above slurry was adjusted to 10.25 ± 0.25 by real-time addition of NaOH solution (1 M). After filtering and washing with pure water until pH ~7, the resultant floccule was homogenized for five passes at 50 MPa with a pressure microfluidizer (NRT model CR5). The viscous product ($\phi_{CNF}$ = 0.2 wt%) was then centrifuged at $10^4$ rpm to remove any un-exfoliated aggregate and stored at 4 °C for further use. The carboxylate content was determined using the conductivity titration method.[6]

Silk NFs were produced by adding poor solvent into aqueous solution of LiBr-regenerated silk fibroin according to the Supplementary Reference 7. Briefly, ethanol was added into the aqueous fibroin solution to achieve the silk concentration of 0.2 wt% and ethanol proportion of 7 vol%. The mixture was incubated at pH 9.5 and 25 ℃ for 2 days to assemble Silk NFs.

Amyloid NFs were fabricated from chicken egg white lysozyme according to the reference.[8] Briefly, lysozyme was dissolved in water (2.0 wt%) and then adjusted to pH 2.0. Fibrillation was performed by incubating at 60 ℃ for 96 h under mildly stirring.

## Supplementary References


1. Deng, J. *et al*. Tunable photothermal actuators based on a pre-programmed aligned nanostructure. *J. Am. Chem. Soc.* **138**, 225–230 (2016).

2. Erb, R. M., Sander, J. S., Grisch, R. & Studart, André R. Self-shaping composites with programmable bioinspired microstructures. *Nat. Commun.* **4**, 1712–1720 (2013).

3. Liu, Y. *et al.* Capillary-force-induced cold welding in silver-nanowire-based flexible transparent electrodes. *Nano Lett.* **17**, 1090–1096 (2017).

4. Jia, Y. *et al*. Surfactant-free emulsions stabilized by TEMPO-oxidized bacterial cellulose. *Carbohydr. Polym.* **151**, 907-915 (2016).

5. Li, X. *et al*. Liquid metal droplets wrapped with polysaccharide microgel as biocompatible aqueous ink for flexible conductive devices. *Adv. Funct. Mater.* **28**, 1804197 (2018).

6. Zhu, L. *et al.* Shapeable fibrous aerogels of metal-organic-frameworks templated with nanocellulose for rapid and large-capacity adsorption. *ACS Nano* **12**, 4462–4468 (2018).

7. Lv, L. *et al.* Biomimetic hybridization of Kevlar into silk fibroin: Nanofibrous strategy for improved mechanic properties of flexible composites and filtration membranes. *ACS Nano* **11**, 8178–8184 (2017).

8. Wu, X. *et al*. Amyloid-graphene oxide as immobilization platform of Au nanocatalysts and enzymes for improved glucose-sensing activity. *J. Colloid Interface Sci.* **490**, 336–342 (2017).
