# Supplementary material for: Evaporation-induced sintering of liquid metal droplets with biological nanofibrils for flexible conductivity and responsive actuation
Source: Nat Commun. 2019 Aug 5;10:3514. doi: 10.1038/s41467-019-11466-5 (PMC6683165; doi:10.1038/s41467-019-11466-5)
Supplement: Supplementary file 3 — Description of Additional Supplementary Files [file 41467_2019_11466_MOESM3_ESM.pdf]

Description of Additional Supplementary Files

File Name: Supplementary Movie 1
Description: Top and smooth mirror-like bottom surfaces of EGaIn layer on glass after evaporation-induced sintering.

File Name: Supplementary Movie 2
Description: Voltage-driven actuating behaviors of Janus film at different voltages (RH ~70%).

File Name: Supplementary Movie 3
Description: Voltage-driven actuating of Janus film simulating predatory motion of cabrites (RH ~70%).

File Name: Supplementary Movie 4
Description: Self-twisting of Janus film driven by NIR radiation (808 nm, 0.8 W cm^{-2} and RH ~70%).

File Name: Supplementary Movie 5
Description: Self-folding of origami driven by NIR radiation (808 nm, 0.8 W cm^{-2} and RH ~70%).

File Name: Supplementary Movie 6
Description: Floating motion of Janus film driven by NIR radiation (808 nm, 0.8 W cm^{-2}) on water surface.
